# Prognostic Factors of First-Time Athletic Patellar Dislocation That Favor Surgical Intervention: A Systematic Review

**DOI:** 10.7759/cureus.80039

**Published:** 2025-03-04

**Authors:** Noopur Ranganathan, Kaya Frazier, James Bicos, Joseph H Guettler

**Affiliations:** 1 Orthopaedic Surgery, Corewell Health William Beaumont University Hospital, Royal Oak, USA

**Keywords:** complications, conservative management patella, lower limb orthopedic surgery, nonoperative, patella dislocation, primary, systematic review

## Abstract

First-time athletic patellar dislocations are either treated surgically with medial patellofemoral ligament (MPFL) reconstruction or conservatively with bracing and physical therapy. Currently, there is no consensus on the best treatment for this condition.

While outcomes are similar between treatment groups, operative treatment has been shown to yield lower redislocation rates and higher Kujala scores. Therefore, the aim of this study was to identify prognostic factors that favor operative treatment of first-time patellar dislocations.

Data sources include PubMed, Cochrane, and Embase. PRISMA-P guidelines for systematic reviews were followed, and a literature search was conducted across three databases based on our search strategy. Articles were imported into Covidence, where they underwent primary screening and full-text review.

After screening 133 potential articles, nine were included in the final review. There were two randomized control trials, six randomized prospective studies, and one non-randomized prospective study. All studies assessed both surgical and conservative interventions for the treatment of first-time athletic patellar dislocations. A total of 548 knees were included with a mode follow-up period of two years. Across all studies, 264 knees (48%) underwent conservative treatment, and 282 knees (52%) underwent operative management. The surgical intervention group had an average redislocation rate of 15.8%, while the conservative intervention group had an average dislocation rate of 35.7%. Identified prognostic factors for surgical intervention include the presence of osteochondral fragments, excessive Q angle, persistent patella subluxation, and patella alta.

Six of the nine studies identified that surgical intervention led to lower redislocation rates and higher Kujala scores, resulting in overall improved outcomes compared to conservative treatment. Identification of the prognostic factors that favor surgical intervention serves to highlight how current treatment guidelines should be modified to avoid future complications of subluxation, patellar instability, or redislocation.

## Introduction and background

First-time patellar dislocations are frequently the result of an acute traumatic injury, most often occurring during sports activities. Patellar dislocations involve the displacement of the patella from the trochlear groove of the femur, most commonly in the lateral direction [1]. Patellar dislocations are frequently observed in active individuals under the age of 20 [1]. Patellar dislocations comprise 2-3% of all knee injuries and occur around six per 100,000 in the general population. These injuries are also present in the pediatric and adult populations, affecting roughly 31 per 100,000 young adults between 10 and 19 years of age [2].

The mechanism of injury is usually a non-contact twisting motion that involves external tibial rotation with the foot planted or a direct impact injury to the medial aspect of the knee [3]. There exists a subset of patients who experience a patellar dislocation as a result of generalized ligamentous laxity or other anatomical deformities that alter the tracking of the patella, such as trochlear dysplasia and patella alta [3]. Other known risk factors for patellar dislocation include patellar hypermobility, increased Q angle, valgus alignment, variations in medial patellofemoral ligament (MPFL) anatomy, and increased femoral antiversion [4-7].

Failure to appropriately treat patellar dislocations can lead to long-term complications of recurrent dislocations, patellar instability, osteoarthritis, and painful subluxation. Traditionally, conservative approaches (non-surgical) to treating first-time patellar dislocations have been used, including cast immobilization, application of a removable splint, knee bracing, taping, and physiotherapy, unless there is an associated presence of either patellar displacement or osteochondral fractures of the lateral femoral condyle [8-10]. However, some studies have suggested conservative management may lead to adverse outcomes, including higher rates of redislocation and lower Kujala scores [11,12]. The Kujala score is a 13-item subjective questionnaire that assesses functional limitations to activities and has been widely used in the field of orthopedics and sports medicine as a measure of patellofemoral knee pain [13]. It is a reliable and valid measure of anterior knee pain and an accurate tool for epidemiological screening within the adolescent female athlete population, although more research is required to assess its applicability to the male athlete population [14].

In recent years there has been a shift towards the utilization of surgical intervention in recent years to treat acute patellar dislocations. Surgical approaches to treat patellar dislocation include MPFL repair, MPFL reconstruction, release of the lateral retinaculum, and medial transfer of the tibial tuberosity [15]. The debate continues over whether first-time patellar dislocations should be treated conservatively or with surgical management. Several studies suggest similar outcomes regardless of the intervention chosen [3,16-20]. Importantly, extant research identifies several factors to consider when deciding between the two interventions, including the mechanism of injury, where the injured knee could have been exposed to an overwhelming situation, or if there exist underlying factors that make the patella inevitable to dislocation. Another measure to identify the optimal treatment modality is the presence of osteochondral or concomitant chondral injuries, which could necessitate operative treatment. Additionally, the potential efficacy of conservative treatment should be evaluated while taking into account the likelihood of patient compliance with the prescribed treatment regimen.

Recent literature suggests operative treatment may yield lower redislocation rates, better clinical outcomes measured by Kujala questionnaire scoring, and increased success in fully returning to sports activity [15,21-24]. There remains a dearth of reliable, large-sample, randomized trials to conclusively state when operative management should be favored over conservative management. Therefore, the aim of this study is to uncover factors that result in improved patient outcomes from operative intervention for first-time athletic patellar dislocations.

Methods

This systematic review was conducted using PRISMA-P guidelines for systematic reviews. A search strategy with keyword search terms was developed to identify articles pertinent to first-time athletic patellar dislocations along with data assessing complication rates and other associated procedural factors. Our search strategy relied on the following keywords: first-time, acute, patellar/patella, dislocation/dislocator, complications, surgery/surgical, and conservative/non-operative. The online databases utilized include PubMed, Cochrane, and Embase.

The population of interest included adult or pediatric patients with first-time patellar dislocations. Interventions evaluated were either conservative or operative treatment. Comparators included factors that influenced receiving one treatment option over the other. Outcomes of interest include Kujala score, return to sport activity, and redislocation rates. Outcomes of interest were compared between the operative and conservative treatment groups.

The identified articles were imported into the Covidence software, an online application tool used for primary screening and data extraction. Once all duplicates were removed, studies were screened based on the defined inclusion and exclusion criteria. Inclusion criteria included publications after 1995, in the English language, and publications that only focused on primary patellar dislocations. Only levels 1 and 2 of evidence were included to ensure high-quality studies. There were no restrictions when conducting the search with regard to publication date, study language, or study type. Systematic reviews were excluded from this analysis. The studies were selected through title and abstract screening by two independent reviewers. All conflicts were resolved by the senior reviewer, and irrelevant studies were removed from the study group. The remaining manuscripts underwent full-text review by two independent reviewers.

Our data extraction forms recorded authorship, year of publication, procedural details, and long-term complications of the study based on our inclusion and exclusion criteria. We recorded the study type, level of evidence, and sample size for the methods of each study. For participants in each study, we noted the year of study, sample size, age of participants, and procedural indication. For the intervention used in each study, we recorded the technique used, factors favoring the procedure and the follow-up period. Lastly, post-procedural complications, incidence of redislocation, and long-term complications were noted for each study.

For data analysis, our data was synthesized to be presented in an efficient manner in tables and figures. The studies are categorized into three groups based on preference for surgery, preference for conservative treatment, or indifference between both treatment options. P-values < 0.05 were considered significant.

## Review

Results

**Figure 1:**
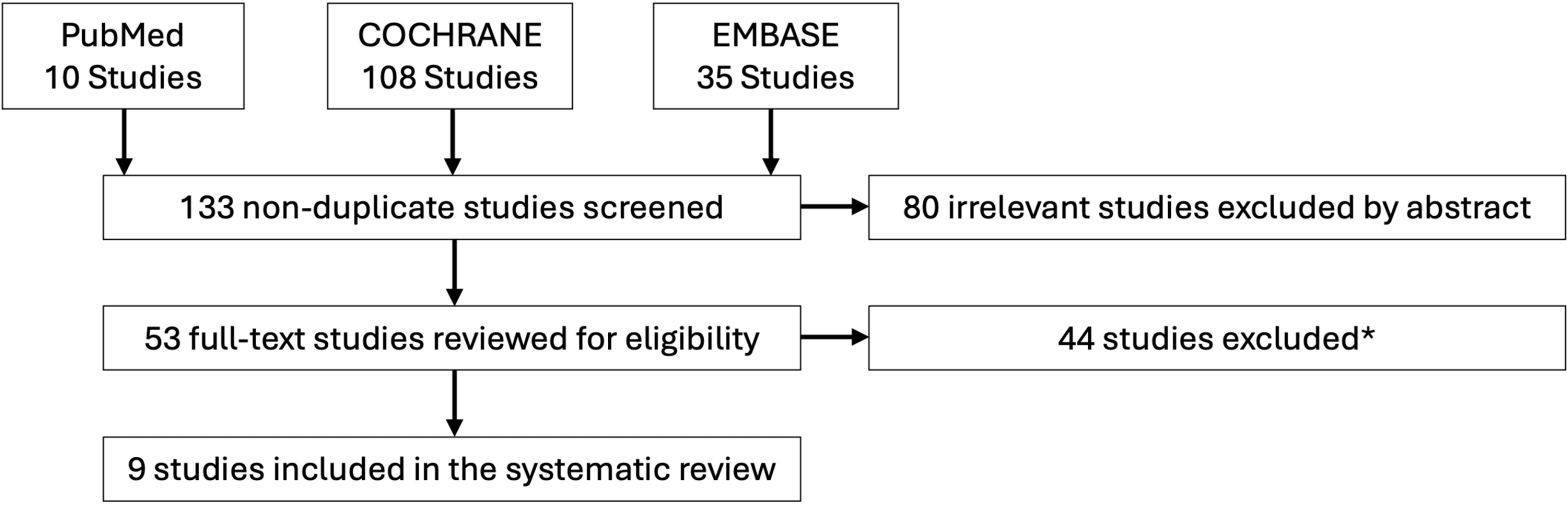
Flowchart of the search strategy based on PRISMA-P guidelines 22 studies were excluded because of non-level 1 or 2 evidence, 10 studies were excluded due to publication year, eight were excluded due to the inclusion of non-primary patellar dislocation, and four studies were excluded due to non-English language.

The study selection PRISMA flow chart diagram is shown in Figure 1. A total of 153 studies were imported for screening, from which 20 duplicate studies were removed. One hundred and thirty-three studies were screened, and 80 studies were excluded based on title and abstract. Fifty-three full-text studies were assessed, and 44 studies were excluded due to a lack of patient-reported data or a lack of both conservative and surgical comparison groups, resulting in nine studies that met our final inclusion/exclusion criteria as represented in Figure 1.

All studies included a comparison of both surgical and conservative management of acute patellar dislocations, complications, and procedural details such as factors favoring operative treatment, outcome parameters measured, Kujala score, redislocation rate, and age at intervention. The modality of conservative treatment was similar across most studies, typically consisting of knee bracing, immobilization and physiotherapy, orthotics, and quadriceps strengthening exercises. The modality of surgical treatment included MPFL reconstruction using the patellar tendon, MPFL repair, medial retinaculum repair, and the Roux-Goldthwait procedure, which corrects a high Q angle similar to an osteotomy of the tibial tubercle without risking compromise of the physis [25]. In addition, many studies had short- and long-term follow-up periods, allowing for the identification of immediate and delayed surgical complications.

Quality assessment was performed on selected studies and displayed in Table 1. We identified two randomized control trials, six randomized prospective studies, and one non-randomized prospective study. Overall, there were nine studies that compared operative versus non-operative treatment of acute patellar dislocations.

**Table 1:** Evaluation of the quality of included studies

Study	Study Design	Level of Evidence	Study Period	Statistical Analysis
Askenberger et al. (2018) [16]	Randomized Control Trial	1	2009-2012	Yes
Bitar et al. (2012) [26]	Randomized Prospective Study	1	2009-2013	Yes
Christiansen et al. (2008) [17]	Randomized Prospective Study	1	2009-2014	Yes
Nietosvaara et al. (2009) [27]	Prospective Cohort	2	2009-2015	Yes
Nikku et al. (1997) [28]	Randomized Prospective Study	2	2009-2016	Yes
Petri et al. (2013) [18]	Randomized Control Trial	1	2009-2017	Yes
Regalado et al. (2016) [29]	Randomized Prospective Study	2	2009-2018	Yes
Sillanpää et al. (2009) [30]	Randomized Prospective Study	1	2009-2019	Yes
Zheng et al. (2019) [32]	Non-Randomized Prospective study	1	2009-2020	Yes

Demographic characteristics were identified and reported in Table 2. All studies assessed both interventions (surgical and conservative) for the treatment of acute patellar dislocations. A total of 548 knees were included with a mode follow-up period averaging two years. Across all studies, 264 knees (48%) underwent conservative treatment while 282 knees (52%) underwent operative management of acute patellar dislocations. Age at the time of intervention ranged from 9 to 40 years of age.

**Table 2:** Demographic characteristics of patients included in the studies

Study	Treatment (Conservative, Surgery, or both)	Number of Knees	Age at Intervention	Follow-Up Period
Askenberger et al. (2018) [16]	Both	74	9-14	2 years
Bitar et al. (2012) [26]	Both	41	9-15	3 years
Christiansen et al. (2008) [17]	Both	77	9-16	4 years
Nietosvaara et al. (2009) [27]	Both	64	9-17	5 years
Nikku et al. (1997) [28]	Both	125	9-18	6 years
Petri et al. (2013) [18]	Both	20	9-19	7 years
Regalado et al. (2016) [29]	Both	36	9-20	8 years
Sillanpää et al. (2009) [30]	Both	40	9-21	9 years
Zheng et al. (2019) [32]	Both	69	9-22	10 years

Patellar redislocation rates were also recorded in Table 3. The surgical intervention group had an average redislocation rate of 15.8%, while the conservative intervention group had an average dislocation rate of 35.7%. Three studies were statistically significant, another two studies did not provide the p-value, and the other four studies did not have statistically significant findings. Complication rates were also reported, with short-term complications most commonly manifesting as superficial wound infections and long-term complications involving redislocations and recurrent subluxations.

**Table 3:** Details of the procedure and rate of patellar redislocation

Study	Conservative Group	Surgical Group	Redislocation in Conservative Treatment (%)	Redislocation in Surgical Treatment (%)	p-value	Complications
Askenberger et al. (2018) [16]	37	37	43	22	0.47	Superficial wound infection: fixation device removed due to discomfort
Bitar et al. (2012) [26]	20	21	35	0	Not provided	Conservative group had higher number of recurrences compared to surgical group
Christiansen et al. (2008) [17]	35	42	20	16.7	Not significant	Recurrent dislocation or subluxation
Nietosvaara et al. (2009) [27]	28	36	71	67	Not provided	Redislocation, recurrent instability
Nikku et al. (1997) [28]	55	70	30	20	0.2	Redislocation, recurrent subluxation
Petri et al. (2013) [18]	8	12	37.5	16.7	0.35	Redislocation
Regalado et al. (2016) [29]	20	16	35	0	0.02	Superficial wound infection; redislocation
Sillanpää et al. (2009) [30]	22	18	29	0	0.02	Redislocation
Zheng et al. (2019) [32]	39	30	20.5	0	0.01	Redislocation

Elements including Kujala scores, outcome parameters, and factors favoring surgery were collected and represented in Table 4. Outcome measures included trochlear dysplasia, lateral patellar tilt, elevated tibial tubercle-trochlear groove distance, patella alta (Caton-Deschamps index), knee injury, and osteoarthritis scores, hyperlaxity, Houghston VAS score, Lysholm II knee score, patellofemoral angle, and Tegner activity scale. Factors that favored surgical treatment were the presence of osteochondral fractures or fragments, a Q angle greater than 20 degrees, the presence of a flat or convex femoral sulcus, persistent subluxation, and Fulkerson classifications I-IV. The surgical intervention group had an average Kujala score of 88.5, which was higher than the conservative intervention group which had an average Kujala score of 82.4 (total score out of 100 points). The difference was statistically significant in three studies, another three studies did not provide the p-value, and the remaining three studies did not observe group statistically significant results.

**Table 4:** Kujala scores and measured parameters *Kujala Score

Study	Outcome Parameters	Factors Favoring Surgery	Conservative Group	Surgical Group	KS* Conservative	KS* Surgery	p-value
Askenberger et al. (2018) [16]	Trochlear dysplasia, Lateral patellar tilt, elevated tibial tubercle-trochlear groove distance, patella alta (Caton-Deschamps index)	Not provided	37	37	95.9	90.9	0.07
Bitar et al. (2012) [26]	Kujala score	Osteochondral fragment	20	21	70.8	88.9	0.001
Christiansen et al. (2008) [17]	Kujala score, Knee Injury and Osteoarthritis Outcome Scores, hyperlaxity, patella dysplasia, trochlear sulcus angle, patella index, meniscus lesions	Osteochondral fragment	35	42	78	85	0.07
Nietosvaara et al. (2009) [27]	Not provided	Q angle greater than 20 degrees, presence of flat or convex femoral sulcus, large femoral osteochondral fracture	28	36	not provided	not provided	not provided
Nikku et al. (1997) [28]	Houghston VAS score, Lysholm II knee score	Persistent subluxation	55	70	not provided	not provided	not provided
Petri et al. (2013) [18]	Kujala score	Not provided	8	12	79.9	88.9	0.17
Regalado et al. (2016) [29]	Patellofemoral angle, Lateral patellar displacement and tilt	Fulkerson types I, II, III, IV	20	16	not provided	not provided	not provided
Sillanpää et al. (2009) [30]	Kujala score, Tegner and Lysholm scale,	Osteochondral fracture, patella alta, excessive Q angle	22	18	90	91	0.82
Zheng et al. (2019) [32]	Kujala score	Patella incapable of being everted to neutral	39	30	80.03	86.27	0.01

Overall results and summative conclusions of each study were extracted and presented in Table 5. Six studies highlighted the benefits of surgical intervention due to lower redislocation rates and higher Kujala scores in the long term. Another two studies reported no significant difference in long-term outcomes, patient satisfaction, and mean Lysholm II knee score between the two groups. The remaining study did not find any significant differences in the redislocation rate between the two groups but reported higher subjective patella stability scores among the surgery group compared to the non-surgical group. Of the six studies that underscored favorable outcomes in patients undergoing surgical intervention, factors that were considered when opting for surgery included the presence of osteochondral fragments, excessive Q angle, patella alta, and patella incapable of being everted to a neutral position.

**Table 5:** Overall outcomes and conclusions of each study

Study	Outcome	Conclusion
Askenberger et al. (2018) [16]	Redislocation rate was significantly lower in the surgical treatment group compared to the conservative treatment group. Operative treatment group had lower scores on the KOOS-Child sport/play and QOL subscales compared to patients treated with a knee brace.	Operative repair of MPFL injury significantly reduced redislocation rate but did not improve subjective or objective knee function compared to conservative treatment of MPFL injury (knee-brace).
Bitar et al. (2012) [26]	Surgical group had higher Kujala scores compared to conservative treatment.	MPFL reconstruction resulted in better results of fewer recurrences and higher Kujala scores compared to conservative treatment group.
Christiansen et al. (2008) [17]	Patella stability subscore was significantly higher in the operative group.	Repair of MPFL does not reduce the risk of redislocation compared to conservative treatment. Only the subjective patella stability score was higher in the MPFL repair group compared to the conservative treatment group.
Nietosvaara et al. (2009) [27]	Long-term functional outcomes after surgery were satisfactory in most patients.	Initial operative repair did not significantly improve long-term outcomes compared to the conservative group.
Nikku et al. (1997) [28]	Functionality was better after closed treatment compared to open treatment.	Surgery and conservative treatments resulted in the same outcomes in terms of patient's overall satisfaction and mean Lysholm II knee score.
Petri et al. (2013) [18]	No significant difference between operative and conservative treatment groups.	Patients with operative treatment had higher Kujala scores and lower redislocation rates compared to patients with conservative treatment.
Regalado et al. (2016) [29]	A higher dislocation rate was identified among the conservative group compared to the operative group.	Slightly better long-term functional outcomes after operative treatment compared to conservative treatment, although both treatment options are feasible.
Sillanpää et al. (2009) [30]	Redislocation rate was significantly lower in the surgical stabilization group compared to the nonsurgical stabilization group.	Patellar stability can be achieved with initial stabilization surgery.
Zheng et al. (2019) [32]	The surgical group had higher Kujala scores compared to conservative treatment. No recurrent dislocation was reported in the surgical group.	Surgical treatment results in better clinical outcomes compared to conservative treatment.

Discussion

This systematic review of published outcomes in first-time athletic patellar dislocations yielded a total of nine studies consisting of two randomized control trials, six randomized prospective studies, and one non-randomized prospective study. While there is no current consensus on whether operative or conservative treatment is the best course of action for first-time patellar dislocations, this systematic review identified factors that favor surgical intervention. The findings align with and expand upon prior research exploring the efficacy, functional outcomes, and long-term implications of both treatment strategies.

Among the randomized controlled trials, Askenberger et al. (2018) conducted a randomized controlled trial where 74 patients were treated with either knee bracing or operative repair of the MPFL. The operative group had significantly lower redislocation rates than the knee bracing group after a two-year follow-up. However, there was no statistically significant difference in subjective and objective knee function between the two groups. Both groups reported similar patient satisfaction with their knee function. Complications included superficial wound infection and a fixation device that was later removed due to discomfort [16]. Petri et al. (2013) further supported surgical intervention in their multicentric, randomized controlled trial in which 20 patients with first-time patellar dislocations were treated either conservatively or operatively. Patients were evaluated 6, 12, and 24 months after their injury, at which time they were presented with a Kujala questionnaire. The operative intervention consisted of soft tissue repairs, including mainly suturing and tightening of the ruptured medial structures. The presence of osteochondral fragments favored surgical treatment. Overall, the surgical intervention resulted in lower rates of redislocation and higher Kujala scores compared to the conservative intervention group [18].

Randomized prospective studies reinforced these findings. Christiansen et al. (2008) directed a prospective randomized study where 77 patients were treated with either isolated MPFL reinsertion or knee bracing. The presence of osteochondral fragments favored surgical intervention. While the patella stability score was significantly higher in the operative group, the study concluded surgical intervention did not significantly reduce the redislocation rate or improve functional outcome based on the Kujala score (two-year follow-up period) when compared to the conservative treatment group [17]. In contrast, Bitar et al. (2012) found that MPFL reconstruction significantly lowered rates of recurrent dislocation and improved Kujala scores, recommending surgical treatment when osteochondral fractures were present. Their study compared the results of operative (MPFL reconstruction) and nonoperative treatment of patellar dislocations via a randomized prospective study. Ultimately, operative management yielded better results in terms of recurrent dislocations or subluxations compared to nonoperative management. Operative treatment also yielded higher Kujala scores after a follow-up period of two years [26]. Nietosvaara et al. (2009) similarly concluded that factors such as a high Q-angle, osteochondral fragments, and femoral sulcus abnormalities favored surgical intervention, although long-term instability rates were comparable between groups. They reported a prospective cohort study in which 64 knees were randomized to receive either operative or nonoperative treatment. Operative treatment consisted of repair of the medial structures of the dislocated patella. After a follow-up period of two years, the authors concluded that initial operative repair had similar long-term outcomes compared to the conservative treatment groups based on recurrent instability rates. Complications included redislocation and recurrent instability [27]. Nikku et al. (1997) found no significant differences using the mean Lysholm II knee score in outcomes or patient satisfaction between operative and conservative management, despite surgical treatment being preferred for patients with osteochondral fragments and persistent subluxation. They conducted a randomized prospective study where 125 patients were either treated surgically or with conservative management of their patellar dislocation. Surgical intervention included repair of the medial retinaculum, while conservative treatment included thigh muscle exercises, cylinder cast, and the use of other orthotics [28]. Regalado et al. (2016) identified a higher redislocation rate in conservatively treated patients, with operative groups showing slightly better functional outcomes. They directed a prospective randomized controlled trial where 36 knees either received operative or conservative treatment. Based on the Fulkerson classification, which served as a prognostic factor for surgical intervention, patients with type I instability underwent lateral retinacula release while types II, III, and IV underwent a modified Roux-Goldwaithe procedure. Conservative management was completed using joint bracing. Both groups underwent the same rehabilitation protocol and were evaluated at 3, 6, 12, and 24 months postoperatively. Patients in the operative group also had slightly better functional long-term outcomes. Complications included a superficial wound infection and redislocation [29]. Sillanpaa et al. (2009) highlighted the long-term benefits of surgical repair, reporting no recurrence of patellar dislocation in the operative group at seven years, whereas six participants in the nonoperative group experienced redislocations. They led a randomized prospective trial in which 40 participants were treated with either bracing or surgical repair of the MPFL. The surgical group had the benefit of having any concomitant injuries to the joint capsule or bony structures of the knee repaired during the index operation, which potentially reduces the risk of early onset of arthritis, continued pain, and joint dysfunction [30].

The only non-randomized prospective study, conducted by Zheng et al. (2019), found that surgical MPFL reconstruction prevented recurrent dislocations and resulted in higher Kujala scores compared to conservative management, reinforcing the advantages of operative intervention in improving clinical outcomes. They led a non-randomized prospective controlled trial where 69 skeletally mature patients were either treated surgically with an MPFL reconstruction or conservatively. The inability to evert the patella to a neutral position favored surgical intervention. The surgical group had no recurrent dislocations and experienced higher Kujala scores (two-year follow-up period) when compared to the conservative treatment group [31].

The collective findings indicate that surgical treatment is particularly beneficial in reducing recurrence, addressing structural abnormalities, and preventing long-term complications such as osteoarthritis. Each subsequent dislocation increases the risk of additional injuries to tendons, ligaments, and bony structures, predisposing patients to early-onset arthritis. While conservative management, including bracing and physical therapy, can achieve similar short-term results, it does not address underlying deformities, which may lead to chronic pain, additional injuries, and decreased activity levels over time. The highlighted studies identified several factors favoring operative intervention, including the presence of osteochondral or chondral injuries necessitating arthroscopy, persistent subluxation of the patella with either MPFL avulsion or disruption, and medial retinacular disruption along with the presence of MPFL disruption. Considering that surgical management has been shown to greatly reduce, if not eliminate, the recurrence of patellar dislocations in a young, athletic population, we believe this review serves as a significant influence in the decision to intervene surgically over conservative management.

With each subsequent dislocation, the risk of additional injury to tendons, ligaments, and other bony structures of the knee increases exponentially and predisposes the patient to develop early-onset arthritis [32]. Additionally, surgical intervention for first-time patellar dislocations has the advantage of addressing injuries to other structures of the knee that may have been damaged during the inciting event, as well as correcting other structural deformities that may have contributed to the dislocation. When treated with bracing and physical therapy alone, these underlying deformities are left unfixed, possibly leading to chronic pain, additional injuries, osteoarthritis, and decreased levels of activity and quality of life in this young population [30]. Therefore, while surgical management and conservative protocols may result in similar outcomes, surgical intervention can prevent or delay the onset of long-term sequelae such as osteoarthritis. Such issues should be considered in the treatment process.

While failing to appropriately treat first-time patellar dislocations can lead to consequences of recurrent dislocations and patellar instability, among others, as previously mentioned, this also leads to increased overall healthcare costs. Previous evidence suggests that operative management or nonoperative therapy with delayed surgical intervention are both cost-effective treatments for first-time patellar dislocations in active adolescents. However, immediate operative management provided the most quality-adjusted life year (QALY) gains across a 10-year time span, suggesting surgical treatment to be the most effective option. The model discussed in this paper by Nwachukwu et al. factored in the return to full activity versus intermediate activity and reported that operative management is not only cost-effective but also has a higher likelihood of restoring patients to full activity, which is another favorable measure for surgical intervention [33].

Other studies have reported that operative management of first-time patellar dislocations decreases the risk of recurrent dislocations in both pediatric and adult populations. A case-control study by Lewallen et al. found that 38.4% of patients treated nonoperatively had recurrent instability, while 51.3% of the same population required surgical intervention after nonoperative management [34]. Another systematic review study by Nwachukwu et al. stated a 31% rate of recurrent patellar instability when treated nonoperatively compared to a 22% rate of recurrence with surgical management, representing additional evidence that supports operative intervention [35].

Although first-time patellar dislocations are the most common knee injury in pediatric and adolescent populations, we were only able to identify nine high-quality studies analyzing the effects of conservative and operative management approaches of patellar dislocations, highlighting the need for further research [36]. Future studies should focus on refining treatment protocols and evaluating long-term outcomes to optimize care for this young, athletic demographic.

Limitations

The main strength of this study is its ability to provide clear evidence supporting operative management in cases where the optimal treatment for first-time patellar dislocations has not yet been determined. However, our study has several limitations. Study outcomes and conclusions are dependent on the quality of available data. Additionally, the small sample size of nine studies was limited by the underreporting of this topic in the literature. Due to the lack of consensus on the management protocol of this condition, there is a need for additional randomized studies evaluating treatment and patient outcomes of patellar dislocations.

Overall, the findings of this study have significant implications for clinical decision-making after first-time patellar dislocation. In pediatric and young adult populations, operative management may be considered the first line of treatment to encourage a faster return to sports activity, augment functional outcomes, and prevent recurrent dislocation, instability, and long-term complications of osteoarthritis.

## Conclusions

This systematic review aimed to identify factors that favored surgical management of first-time athletic patellar dislocations. Our systematic review consisted of randomized controlled trials, randomized prospective studies, and non-randomized prospective studies.

Based on the selected studies, we conclude that the factors that suggest improved outcomes from operative management of first-time athletic patellar dislocations include the presence of osteochondral or chondral injuries and fragments, excessive Q angle, patella alta, persistent patellar subluxation with MPFL disruption or avulsion, medial retinacular disruption with MPFL disruption, and patella incapable of being everted to a neutral position. Six of the nine studies found that surgical intervention yielded lower redislocation rates and higher Kujala scores, ultimately suggesting that not all first-time patellar dislocations are created equally, unlike current treatment guidelines indicate.
